# Identification of sex-associated network patterns in Vaccine-Adverse Event Association Network in VAERS

**DOI:** 10.1186/s13326-015-0032-2

**Published:** 2015-08-19

**Authors:** Yuji Zhang, Puqiang Wu, Yi Luo, Cui Tao

**Affiliations:** Division of Biostatistics and Bioinformatics, University of Maryland Greenebaum Cancer Center, Baltimore, USA; Department of Epidemiology and Public Health, University of Maryland School of Medicine, Baltimore, USA; University of Wisconsin-Madison, Madison, Winsconsin USA; Department of Computer Science and Engineering, Lehigh University, Bethlehem, Pennsylvania USA; School of Biomedical Informatics, University of Texas Health Science Center at Houston, Houston, Texas USA

## Abstract

**Background:**

Vaccines are one of the most important public health successes in last century. Besides effectiveness in reducing the morbidity and mortality from many infectious diseases, a successful vaccine program also requires a rigorous assessment on their safety. Due to the limitations of adverse event (AE) data from clinical trials and post-approval surveillance systems, novel computational approaches are needed to organize, visualize, and analyze such high-dimensional complex data.

**Results:**

In this paper, we proposed a network-based approach to investigate the vaccine-AE association network from the Vaccine AE Reporting System (VAERS) data. Statistical summary was calculated using the VAERS raw data and represented in the Resource Description Framework (RDF). The RDF graph was leveraged for network analysis. Specifically, we compared network properties of (1) vaccine - adverse event association network based on reports collected over a 23 year period as well as each year; and (2) sex-specific vaccine-adverse event association network. We observed that (1) network diameter and average path length don’t change dramatically over a 23-year period, while the average node degree of these networks changes due to the different number of reports during different periods of time; (2) vaccine - adverse event associations derived from different sexes show sex-associated patterns in sex-specific vaccine-AE association networks.

**Conclusions:**

We have developed a network-based approach to investigate the vaccine-AE association network from the VAERS data. To our knowledge, this is the first time that a network-based approach was used to identify sex-specific association patterns in a spontaneous reporting system database. Due to unique limitations of such passive surveillance systems, our proposed network-based approaches have the potential to summarize and analyze the associations in passive surveillance systems by (1) identifying nodes of importance, irrespective of whether they are disproportionally reported; (2) providing guidance on sex-specific recommendations in personalized vaccinology.

**Electronic supplementary material:**

The online version of this article (doi:10.1186/s13326-015-0032-2) contains supplementary material, which is available to authorized users.

## Background

Vaccines are one of the most cost-effective public health interventions to date, leading to at least 95–99 % decrease of most vaccine-preventable diseases in the United States [1]. While their benefits far overweigh their risks and costs, vaccines are accompanied with specific adverse events (AEs). Assessment of vaccine safety usually starts at the pre-approval stage, when information about AEs is collected during Phase I-IV of clinical trials. However, there are several limitations of such information. First, clinical trials usually have small sample sizes which are insufficient to detect rare AEs. Second, clinical trials are usually carried out in well-defined, homogeneous populations within relatively short follow-up periods, which may limit the generalizability of their effect in all populations. Therefore, the complete safety profiles associated with a vaccine cannot be fully established only through clinical trials. Post-approval surveillance of vaccine AEs is needed to assess the vaccine safety throughout its life on the market.

The Vaccine AE Reporting System (VAERS) is a passive surveillance system to monitor vaccine safety after the administration of vaccines licensed in the United States [2]. The VAERS is co-managed by the United States Food and Drug Administration (FDA) and the Centers for Disease Control and Prevention (CDC). By the end of 2013, the VAERS contains more than 200,000 reports in total, including 72 vaccine types and 7368 reported symptoms/AEs. However, there are several limitations we need consider in the analyses of spontaneous reporting systems such as VAERS, including lack of verification of reported diagnoses, lack of consistent diagnostic criteria for all cases with a given diagnosis, wide range of data quality, underreporting, inadequate denominator data, and absence of an unvaccinated control group [3]. To address some of these limitations, various data mining approaches have been developed to identify potential signals in the data [4]. Most of these approaches focus on disproportionality of reporting, which aims to identify conditions that comprise a larger proportion of reported events for a given vaccine, compared to other vaccines in the same reporting system [3]. However, such disproportionality methods still have difficulties to identify potential vaccine-AE associations due to the limitations of VAERS data. In Bate et al. 2009 [5], the authors suggested that a single drug-AE should be analyzed in the context of all drug-AE associations. Harpez et al. proposed a clustering approach to identify drug groups that were reported to have same AEs [6]. However, this approach didn’t account for all co-administered drugs and co-occurring AEs. Since VAERS receives more than 14,000 reports every year, there is a pressing need to develop novel approaches to organize these high-dimensional VAERS data and identify potential vaccine-AE associations.

In recent years, network analysis emerges as a very promising approach for simultaneous representation of complex high dimensional data. Specifically, these network-based computational approaches gained popularity and have become a new paradigm to investigate associations among biological entities (e.g., drugs, diseases, and genes). Applications of these approaches include drug repositioning [7, 8], disease gene prioritization [9–11], and identification of disease relationships [12, 13]. These network analysis approaches are usually developed based on the observations from real-world networks. First, most real-world networks (e.g., WWW network, protein-protein interaction network, and social network) are not randomly organized but are driven by preferential attachment and growth (e.g., some nodes have more connections than others). Such networks are called “Scale-free” networks. In the “scale-free” network, the most highly connected nodes are called “hub’ nodes. Second, most real world networks are modular, comprised of small, densely connected groups of nodes. Network analysis metrics and algorithms have been designed to identify network hub nodes and modules in a scale-free network. Ball and Botsis proposed a network-based approach to aid visualization of patterns in VAERS data that a medical expert might recognize as clinically important [14]. In our previous work, we developed a network analysis approach to dentify vaccine-related networks and their underlying structural information from PubMed literature abstracts, which were consistent with that captured by the Vaccine Ontology (VO) [15]. The modular structure and hub nodes of these vaccine networks reveal important unidentified knowledge critical to biomedical research and public health and to generate testable hypotheses for future experimental verification.

In this paper, we proposed a network-based approach to investigate the vaccine-AE association network from VAERS data. First, we extracted and represented data summarized from VAERS database using Resource Description Framework (RDF). We calculated overall proportional reporting ratio (PRR), yearly PRR and sex-specific PRR for each vaccine-AE association in the VAERS. We then applied a series of network approaches to the network consisting of significant vaccine-AE associations (i.e., PRR > 1). Specifically, we compared network properties of (1) vaccine-AE association network based on reports collected over a 23 year period as well as each year; (2) sex-specific vaccine-AE association network. We observed that (1) network diameter and average path length don’t change dramatically over a 23-year period, while the average node degree of these networks changes due to the different number of reports during different period of time; (2) vaccine-AE associations derived from different sexes show sex-associated patterns in sex-specific vaccine-AE association networks.

The rest of the paper is organized as follows. In Section Materials and methods, we introduce our methodology on data collection, summarization, representation, and analysis. In Section Results, we present the result of our study. In Section Discussions, we discuss the potential scientific contributions of this study. In Section Conclusions and future work, we conclude the paper and discuss future directions.

## Materials and methods

**Fig. 1:**
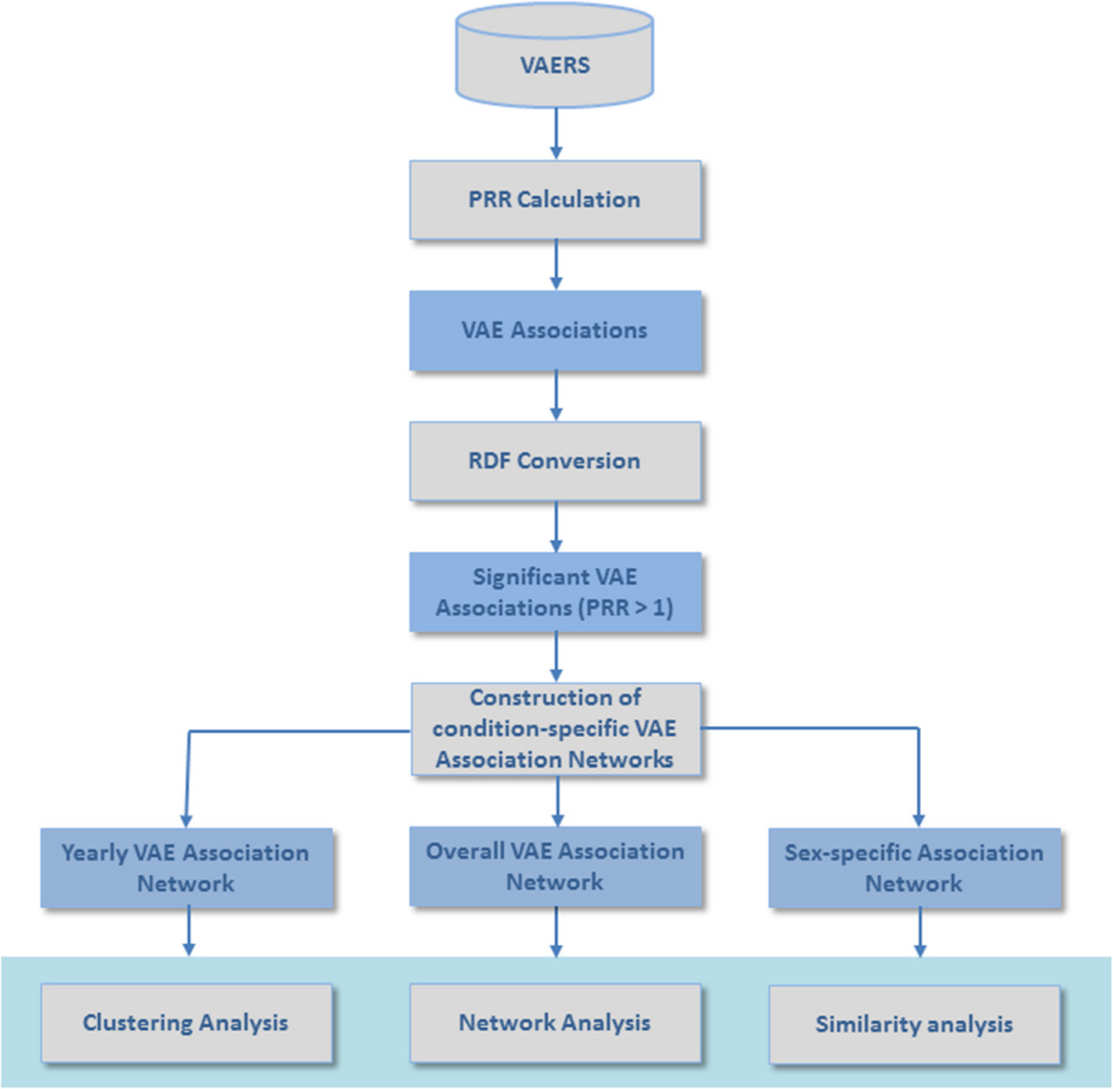
Overview of the proposed study. VAERS: Vaccine AE Reporting System; RDF: Resource Description Framework; VAE: Vaccine-AE; PRR: Proportional Reporting Ratio

In this section, we first describe the data resources and preprocessing method in this work. We then introduce our proposed network-based approach for investigating vaccine-related associations derived from VAERS. Figure 1 illustrates the steps of the proposed approach.

### VAERS database

We downloaded raw data from the VAERS system in comma-separated value (CSV) format (https://vaers.hhs.gov/data/data). All the data from 1990–2013 was loaded to a mySQL relational database for further processing. The VAERS database contains three tables: Data, Data sources and preprocessing Symptom, and Vaccine. The Data table contains general information about each report including VAERS report ID, date the report was received, the state patient was in, age and sex of the patient, and detailed description of the symptom (e.g., if the symptom was life threatening, if the patient in the report died and if-so the date of death, if the patient ever attend the ER for treatment, and if so, how many days was the patient administered at the hospital.). The Symptom table contains a list of symptom terms (MedDRA terms) involved in the report. Completed information about one report can be jointed from the three tables using VAERS ID. The Vaccine table includes information about the vaccine administered to the patient such as vaccine manufacturer, type of vaccine, dosage of the vaccine, vaccination route, vaccination site, and vaccination name.

### Statistical summary of VAERS data

As we discussed above, the VAERS is a spontaneous reporting system which contains unverified reports with inconsistent data quality. Symptoms reported occurring after vaccination do not necessarily indicate a causality association with the vaccine. Therefore, we used statistical methods to summarize meta-level features of vaccine-symptom pairs. For each vaccine-symptom pair, we calculated the following features (1) the number of reports that contains the pair; (2) the number of reports that contains the pair each year; (3) the demographic distribution among the reports that contain the pair (total and yearly) grouped by gender and age groups; and (4) overall proportional reporting ratio (PRR) and yearly PRRs [16]. A PRR is the ratio between the frequency with which a specific symptom (e.g., AE) occurs for a vaccine of interest (relative to all symptoms reported for the vaccine) and the frequency with which the same symptom occurs for all vaccines reported to the VAERS (relative to all symptoms for all vaccines reported to VAERS) [3]. A PRR greater than 1 suggests that the post-vaccination symptom (AE) is more commonly observed for individuals administrated with the particular vaccine, relative to all other vaccines reported to the VAERS.

The overall PRR ratio of a vaccine (*V*) and a symptom (*S*) association was calculated by (Num_reports for *V* that contains*S*_/Num_all thereports for *V*_)/(Num_totalreports that contains S_/Num_total reports in VAERS_).

The yearly PRR ratio of a vaccine (*V*) and a symptom (*S*) association in Year (*Y*) was calculated by (Num_reports for *V* that contains *S*in year Y_/Num_all the reports for *V* in Year *Y*_)/(Num_totalreports that contains S in Year *Y*_/Num_totalreports in VAERS in Year *Y*_).

The sex-specific PRR ratio or a vaccine (*V*) and a symptom (*S*) association in Gender (*G*) was calculated by (Num_reports for *V* that contains *S for*patient with *G*_/Num_all thereports for *V* for patient with *G*_)/(Num_totalreports that contains *S for* patient with *G*_/Num_totalreports in VAERS for patient with *G*_).

### RDF conversion

The statistical summary introduced in the previous section was stored in a relational database and converted to the Resource Description Framework (RDF) format. We have introduced detailed information about how to represent vaccine symptom pairs with meta-information in RDF and our vision on linking heterogeneous vaccine-related data sets using linked data approach in our previous work [17].

**Fig. 2:**
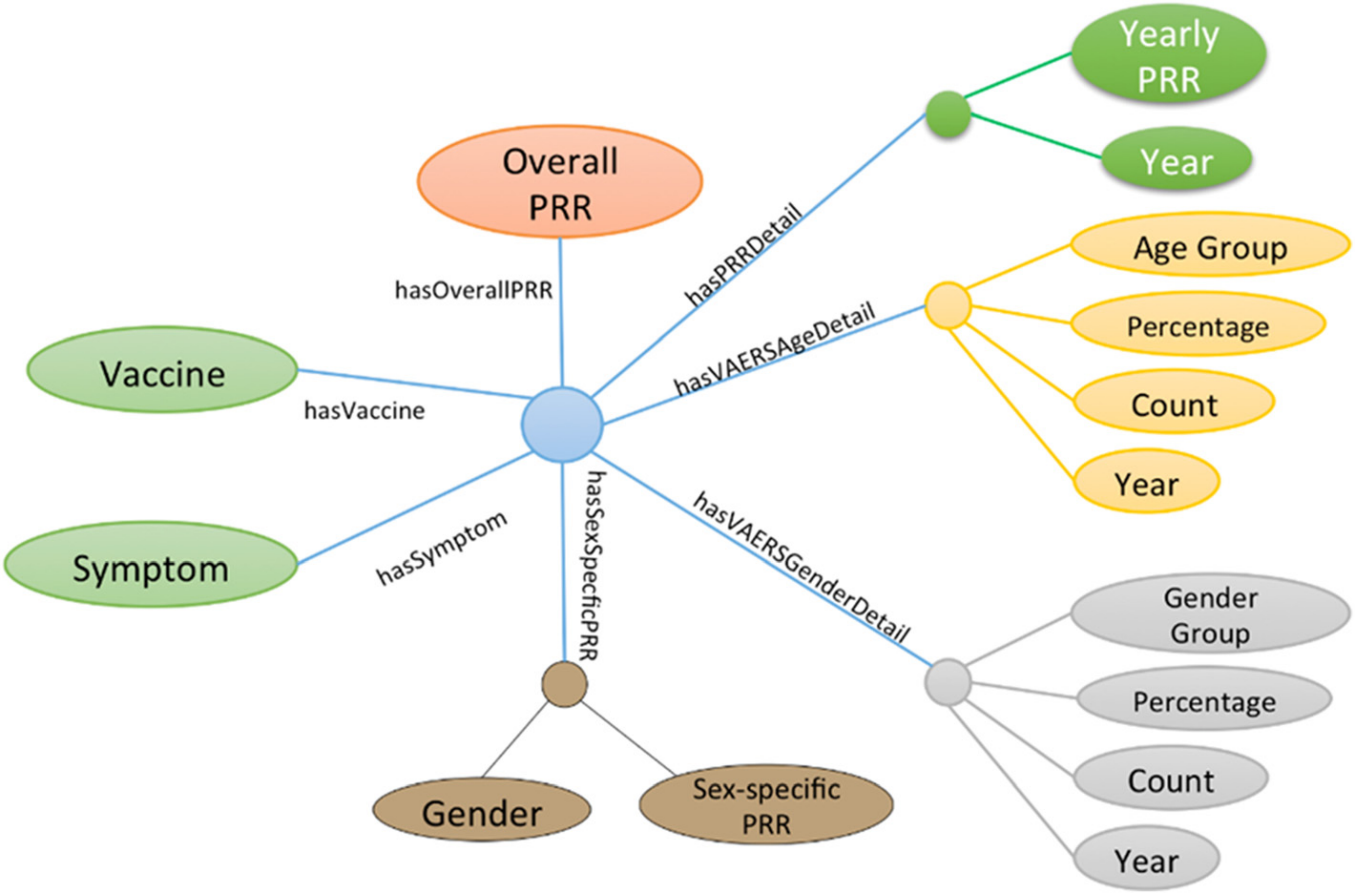
Sample RDF graph representation of vaccine AE association

Figure 2 shows the meta-level RDF graph representation of a vaccine symptom association. Each unique association (vaccine-symptom pair) has an unique identifier. The corresponding vaccine, symptom, demographic distribution, and PRR values are also represented in RDF. SPARQL queries can be conducted to retrieve useful information for network analysis which we will introduce in the next section.

### Network analysis

The analysis of network properties was performed using the “Network Analyzer” plugin in Cytoscape [18]. Cytoscape is an open-source platform for integration, visualization, and analysis of biological networks. Its functionalities can be extended through Cytoscape plugins. Scientists from different research fields have contributed more than 160 useful plugins so far. These comprehensive features allow us to perform thorough network-level analyses, visualization of our association tables, and integration with other biological networks in the future. In this study, the average node degree, average path length, and network diameter of one network was calculated.

The vaccine-AE association network is a bipartite network, which consists of interactions between two different types of nodes (X-type and Y-type), with edges connecting only nodes of different types. To calculate the similarity of one type of nodes (e.g., X-type nodes) based on their interactions with another type of nodes (e.g., Y-type nodes), the Pearson correlation coefficient (PCC) was employed as an association index. We assume that node A and B are X-type nodes, and the PCC between node A and B is calculated by

1 $$ PC{C}_{AB}=\frac{\left|N(A)\cap \mathrm{N}(B)|\cdot {\mathrm{n}}_y-\left|N(A)\right|\cdot \Big|N(B)\right|}{\sqrt{\left|N(A)\right|\cdot \Big|N(B)\Big|\cdot \left({n}_y-\left|N(A)\right|\right)\cdot \left({n}_y-\left|N(B)\right|\right)}} $$

where A and B are nodes of same type, *N(A)* and *N(B)* are their total number of interactions with A and B, *N(A)∩N(B)* is the total number of Y-type nodes that interact with both A and B, and *n*_*y*_ is the total the total number of Y-type nodes in the network. A PCC of 1 indicates a perfect overlap, 0 corresponds to the number of shared interactors expected by chance and −1 depicts perfect anti-correlation.

The hierarchical clustering analysis is used to identify the similarities among vaccines using their association indexes. Both the heatmap of the dendrogram are used to visualize the clustering results. The clustering analysis and visualization of vaccine-AE association network was performed using the GAIN tool [19].

## Results

### Overview of the results

Overall, we extracted 2,346,367 vaccine-AE associations from the VAERS system, with 83,148 distinct associations. We defined that a vaccine-AE association is significant if PRR for this association is greater than 1. Among all vaccine-AE associations reported in the VAERS, we identified 277,698 vaccine-AE associations, 53,795 of which have overall PRR greater than 1 between 1990 and 2013. We also investigated yearly PRRs of these associations. For one specific year, we define that a vaccine-AE association is significant if the yearly PRR is greater than 1. Table 1 presents the numbers of significant associations for each year (*N*_*link*_ Column).

**Table 1:** General characteristics of the networks

	N_node_	N_link_	Average degree	Average path length	Network diameter
1990	342	990	5.79	3.05	7
1991	634	2,756	8.69	2.69	5
1992	553	2,275	8.23	2.58	5
1993	517	2,263	8.75	2.45	5
1994	549	2,487	9.06	2.46	5
1995	637	3,080	9.67	2.54	6
1996	628	3,028	9.64	2.54	5
1997	660	3,270	9.91	2.51	5
1998	793	3,989	10.06	2.54	5
1999	1,047	5,245	10.21	2.67	5
2000	1,065	5,708	10.72	2.59	5
2001	906	4,904	10.83	2.53	5
2002	987	5,289	10.72	2.58	5
2003	1,430	8,197	11.46	2,52	5
2004	1,160	6,491	11.19	2.62	5
2005	1,315	7,290	11.08	2.66	6
2006	1,978	12,128	12.26	2.66	5
2007	3,103	20,214	13.03	2.59	5
2008	3,045	20,196	13.27	2.58	6
2009	3,351	22,054	13.16	2.57	5
2010	2,639	18,041	13.67	2.62	5
2011	2,142	13,533	12.64	2.69	5
2012	1,943	12,292	12.65	2.72	5
2013	826	4,886	11.83	2.72	5
Female	4,947	49,616	20.14	2.35	5
Male	4,519	51,578	22.83	2.35	5
Overall	5,938	53,742	18.10	2.48	5

Based on the significant yearly or overall associations, we further investigated these association networks using different network properties. Table 1 presents the general characteristics of the overall association network as well as yearly-significant association networks, including average node degree, average path length and network diameter. This demonstrates that vaccine-AE network is dense network, with any given node connected to all other nodes through an average of approximately two other nodes and a maximum of 5–6 nodes. It is explained partly by that many vaccines are co-administered. However, given that there are more AEs than vaccines in the network, it is plausible that many AEs were reported together. Another interesting observation was that across 23 years, the average path length and network diameter for yearly vaccine-AE association networks don’t change dramatically. It indicated that in most cases, it is relatively common that two vaccines sharing one AE or two AEs associate with one vaccine in the network. On the other hand, the average node degree of these networks changes over time, partly due to the increasing number of reports received from 1990 to 2013 (Table 1). All the network information in Table 1 were presented in Additional file 1.

### Different AE association patterns in different genders

**Fig. 3:**
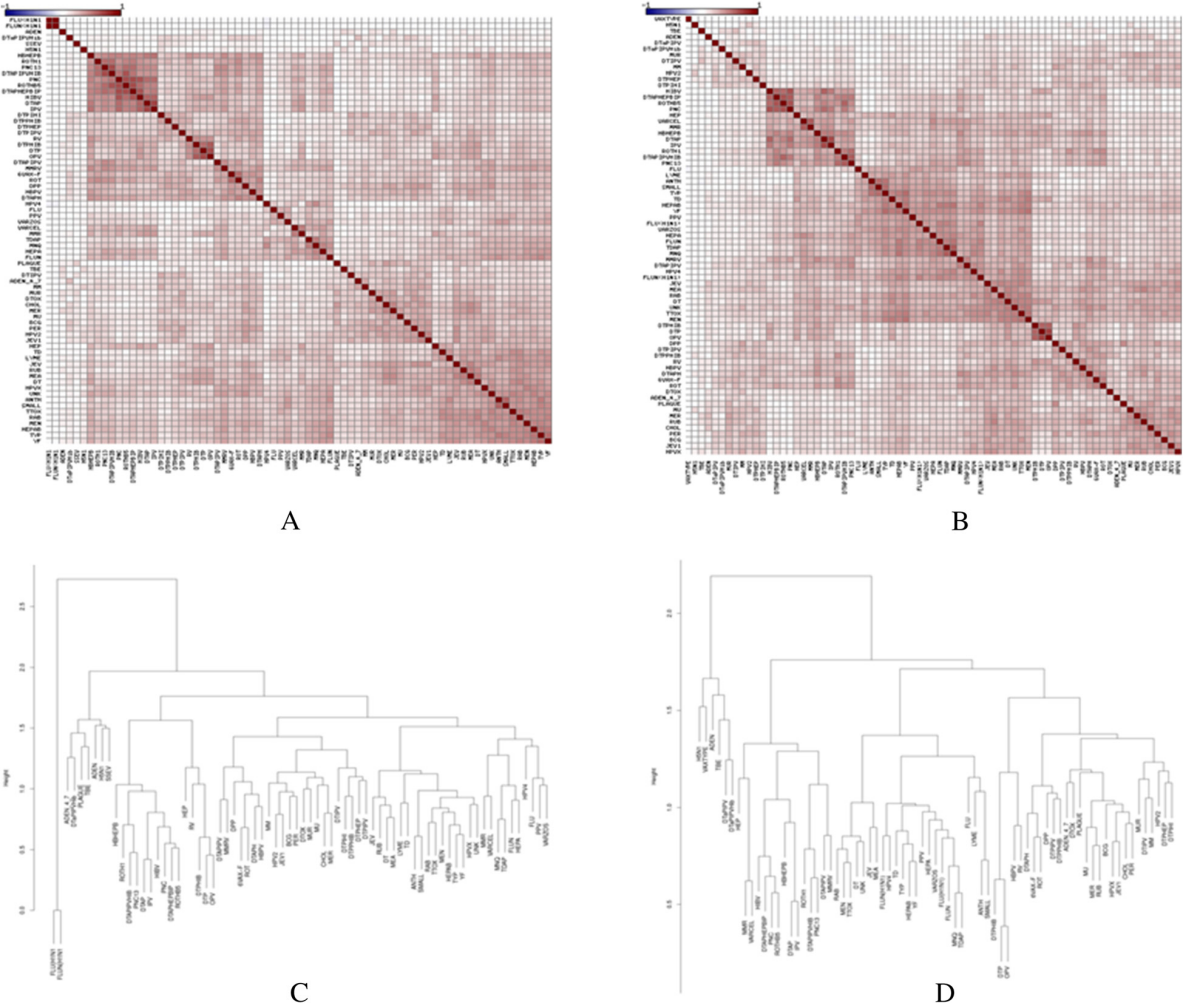
Comparison of vaccine similarity in different sexes. **a** Hierarchical analysis of vaccines based on association information in female reports; **b** Hierarchical analysis of vaccines based on association information in male reports; **c** Dendrogram of vaccine similarity in female reports; **d** Dendrogram of vaccine similarity in male reports

We further investigated whether vaccine-AE associations are different between genders. We constructed sex-specific vaccine-AE association networks by computing the PRR based on reports only from female/male populations. There are 49,616 and 51,578 significant vaccine-AE associations (i.e., PRR > 1) in female and male populations, respectively. The network properties of these two sex-specific association networks are similar with overall association network (Table 1). We clustered the vaccines based on their association indexes calculated by their associations with AEs. In Fig. 3a and b, we observed different similarity patterns in female (Fig. 3a and male (Fig. 3b). For instance, HBHEPB, ROTH1, PNC13, DTAP IPVHIB, PNC, ROTHB5, DTAPHEPBIP, HIBV, DTAP, and IPV were clustered together based on their associations with adverse events in the female population. Besides most of the vaccines that were grouped in the female population, we also found four more vaccines in the same group in the male population, including PNC, HEP, VARCEL, and MMR. Similarly, while DIPHIB, DTP, and OPV were tightly clustered in the male population, RV was also grouped in this cluster in the female population. The dendrograms indicate the same differences between two populations (Fig. 3c and d). These results indicate that there are indeed sex-specific reponse differences after vaccine injection.

**Fig. 4:**
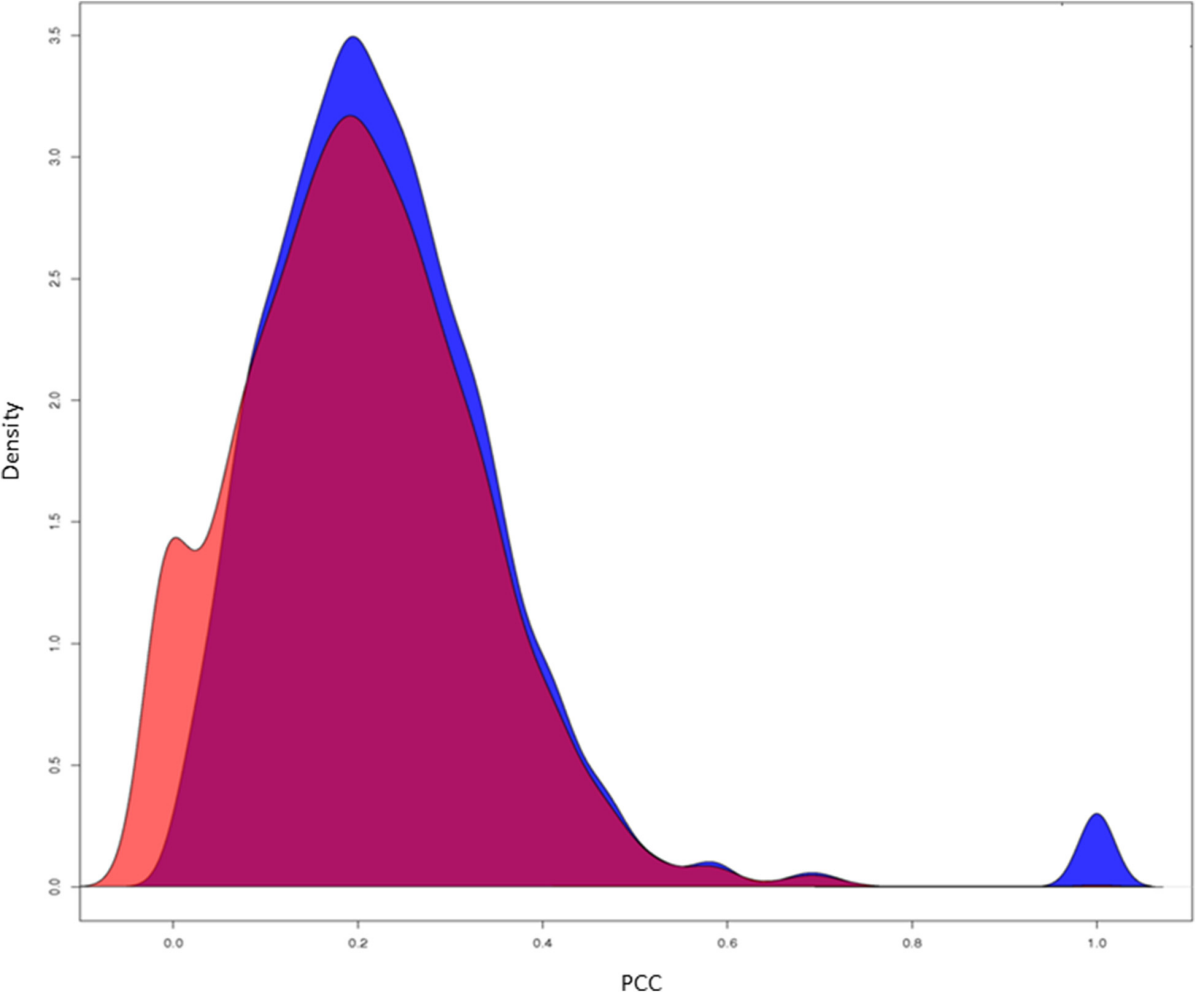
Density plot of PCC association indexes in female vs male populations (red: female; blue: male)

We also compared whether pairs of vaccines with similar association profiles in female-specific association network are also similar in male-specific network. In Fig. 4, the distributions of PCC indexes are different in two populations, indicating that there are some sex-specific associations in both populations, although majority of association relationships can be identified in both populations. Specifically, there were more vaccine pairs showing high similairties in the male population than in the female population. The underlying mechanisms need further investigation using other types of biological data, such as genomic, metablomic, and proteomic level measurement data.

## Discussions

Most vaccine-preventable diseases have declined in the United States by at least 95–99 % [20, 21]. However, vaccines are pharmaceutical products that carry risks. Certain biomarkers or individual variations could implicate different vaccine responses, which are essential for precision medicine. Identifying these associations is critical to vaccine safety, which reassures public acceptance of vaccines. One way to address this question is the post-approval surveillance of vaccine AEs. For instance, the VAERS is a passive surveillance system to monitor vaccine safety after the administration of vaccines licensed in the United States [2]. Such surveillance data can complement the original safety evaluation data generated from the clinical trial phases and provide more comprehensive safety assessment in a much larger population. We are one of the first research groups that investigates sex-specific vaccine-AE association patterns by integrating traditional statistical signal detection and network analysis approaches. Our findings indicated that saftety signals present different patterns in female and male population. This is consistent with previous studies in the vaccine community [22, 23]. With high-throughput technology advances such as next generation sequencing, transcriptomics, epigenetics, proteomics, and new computational approaches to interpreting big data, we expect a better understanding of associations and mechanisms of vaccine AEs and immunogenicity. Network analysis approaches is one of the promising stratetigies to integrate such heterogeneous “big data”, leading to a more personalized or individual approach to vaccine practice in the near future.

## Conclusions and future work

In this paper, we proposed a network-based approach to investigate the vaccine-AE association network from VAERS. The results indicated that (1) network diameter and average path length of vaccine-AE association networks don’t change dramatically over a 23-year period, while the average node degree of these networks changes due to the different number of reports during different period of time; (2) vaccine-AE associations derived from different genders show sex-associated patterns in sex-specific vaccine-AE association networks. To our knowledge, this is the first time that a network-based approach has been used to identify sex-specific association patterns in a spontaneous reporting system database. Due to unique limitations of such passive surveillance systems, network-based approaches have the potential to (1) identify nodes of importance, irrespective of whether they are disproportionally reported; (2) provide guidance on sex-specific recommendations in personalized vaccinology.

Extensions of this work include: (1) integration of other spontaneous reporting system databases (e.g., the European Adverse events following immunization (AEFI) system) to construct more complete vaccine-AE association networks; (2) incorporation of other complementary public databases such as Semantic MEDLINE [24]; (3) development of advanced network-based approaches taking the PRR values into account; (4) investigation of other types of data mining methods to assess the significance of vaccine-AE associations; (5) focused investigation of examples based on the network parameters; and (6) identification of sex-specific subnetwork patterns of AE correlation networks.

## Additional file

Additional file 1:
**Network file containing all the networks presented in Table** **1**. (CYS 13422 kb)
